# Many Pros and a Little Cons: Experiences of First-Time Guide Dog Recipients

**DOI:** 10.3390/ani15162461

**Published:** 2025-08-21

**Authors:** Chalotte Glintborg, Johan Trettvik, Rasmus Holm, Tia G. B. Hansen

**Affiliations:** 1 Department of Communication and Psychology, Center for Developmental & Applied Psychological Science (CeDAPS), Aalborg University, Teglgaards Plads 1, 9000 Aalborg, Denmark; trettvik@ikp.aau.dk; 2 Independent Scholar, 4000 Roskilde, Denmark; rholm17@student.aau.dk; 3 Department of Communication and Psychology, Center for Human Animal Psychology (CHAP), Aalborg University, Teglgaards Plads 1, 9000 Aalborg, Denmark; tia@ikp.aau.dk

**Keywords:** guide dogs, visual impairment, blindness, disability aids, qualitative study

## Abstract

Guide dogs are primarily seen as mobility aids for people with visual impairments, but they may also influence users’ psychological well-being and social lives. Benefits of being partnered with a guide dog have been found in several countries but Scandinavian studies are sparse. This qualitative study explores the lived experiences of six Danish guide dog partners through semi-structured interviews. A reflexive thematic analysis found six primary benefits: improved mobility, increased physical activity, enhanced freedom and independence, new social interactions, a strong emotional bond with the dog, and better mental well-being. Users also reported challenges, including frequent distractions caused by the public interacting with the dog, difficulties during the initial adjustment period, increased cleaning, and occasional trouble finding care for the dog. Participants unanimously emphasized the irreplaceable value of their guide dogs and positive socioemotional dimensions of guide dog partnerships. The findings add Scandinavian voices that support existing international research and note three areas of potential improvement: better public education about guide dog etiquette, enhanced support during the adjustment period, and improved care-sharing arrangements. The study underscores the importance of considering guide dogs as both functional aids and sources of emotional and social support.

## Simple Summary

Guide dogs are trained to help people who are blind or severely visually impaired. Previous research has provided rich literature on the subject, but little is known about first-hand experiences of guide dog users in Scandinavia. This study explored how Danish guide dog users perceive benefits and challenges of having a guide dog. Six individuals participated in in-depth interviews. The results show that guide dogs improve users’ mobility, physical activity, independence, social interactions, mental well-being, and overall quality of life. Users also form a close emotional bond with their guide dogs. However, some challenges were reported, including members of the public interfering with working guide dogs, difficulties during the initial adjustment period, increased cleaning needs, and finding dog care when traveling. Despite challenges, all participants were highly satisfied with their guide dog, and we suggest that most challenges could be addressed better. The findings highlight the broad and meaningful impact of guide dogs beyond their role as mobility aids.

## 1. Introduction

Visual impairment can impact a person’s ability to move freely and impose practical constraints. It also makes social interactions more difficult, which may lead to feelings of isolation and exclusion [1]. Being visually impaired is generally associated with elevated risk of mental challenges such as an increased incidence of depression [2,3], a reduced ability to cope with daily activities [4], and a decreased quality of life [5]. It has been reported that persons with visual impairments have significantly lower quality of life compared to some other chronic conditions such as deafness [6]. In Denmark, the context of the current study, the most recent report from the Health Ministry funded Danish Center for Social Science Research found that blind and severely visually impaired individuals still face some barriers to mobility and education. Compared to people without disabilities, they also had poorer physical health and a higher incidence of loneliness but reported little decrease in quality of life [7].

A guide dog may be a solution to some of the difficulties. Having a guide dog can improve quality of life and enhance social relationships, thereby reducing the risk of mental challenges [8]. A guide dog is specially trained to assist blind and severely visually impaired individuals [9]. In addition to being a physical aid, a guide dog is also a unique, emotional, and well-trained companion that accompanies a person wherever they go [10].

This human–animal partnership represents a unique form of interspecies collaboration where the welfare of both species becomes intrinsically linked. Understanding guide dog experiences requires examining not only human benefits but also how canine welfare is taken into account and the quality of the human–animal bond that develops.

Internationally, the number of active guide dogs declined by 9% from 2019 to 2022, while the number of guide dogs trained each year decreased by 22% [11]. One factor could be that medical improvement now enables treatment of some of the diseases that previously led to age-related blindness [12], but not all causes can be eliminated. Currently about 32,000 Danes live with visual impairment, and approximately 12,500 of these have less than 10% eyesight and meet the membership criteria for the country’s oldest and largest association for people with visual impairment, the Danish Society for the Blind [12,13]. In Scandinavian welfare state systems, most medical procedures and many personal aids that are necessary to compensate for functional impairments are tax-paid, and in Denmark, guide dogs can be granted as a personal aid under the Social Services Act §112. The dogs and their training are bought from private providers, which are primarily two NGOs, Danish Society for the Blind and Users’ Guide Dog Association.

Users’ Guide Dog Association has trained about 35 of the active guide dogs [14]. This organization estimates that about 1800 Danes have severe enough visual impairments to apply for a guide dog. However, only about 235 guide dogs are currently active, according to Danish Society for the Blind, which delivers 20–25 new guide dogs each year [15]. The discrepancy between potential and actual guide dog recipients suggests barriers to access and service provision that warrant further investigation.

Users’ first-hand perspectives on benefits and challenges are important, and qualitative analysis of in-depth interviews is a main road to such exploration. While such contributions exist from several anglophone countries and a few others, they are absent from the sparse Scandinavian literature on guide dogs [7,12,16,17,18]. Users’ own voices may appear in testimonials and reports from guide dog providers, e.g., [14], but these may be biased towards advantages, and independent research is missing.

We therefore conducted an interview study of Danish guide dog users, using the following research questions:

1. What benefits do guide dog users experience from having a guide dog?
2. What challenges and disadvantages do guide dog users face from having a guide dog?

The aim of the study is to describe user experiences with guide dogs, discuss how they align with the existing international literature, and suggest areas of potential improvement.

### Previous Research

A report from a Danish guide dog provider (Users’ Guide Dog Association, [14]) asserted, among other things, that having a guide dog contributed to a greater sense of freedom, improved mental well-being, and allowed users to engage more in society—especially due to increased energy in daily life and greater safety in traffic. That is also found in international research, at least partly.

A British study [8] found that having a guide dog can improve a person’s quality of life, while individuals on a waiting list for a guide dog experienced a decrease in their quality of life over a six-month period. However, an Austrian study [19] did not find a higher quality of life among guide dog users compared to non-users. On the other hand, their participants did report that the guide dog increased their personal independence and positively impacted their physical health.

Regarding the general benefits of living with a dog, guide dog users often experience the same psychological and social advantages as the general population. These benefits can be more pronounced in guide dog users because they spend much time together and typically develop a working relationship with the dog, which often leads to a strong bond [1]. Although a guide dog is primarily a mobility aid, many users report that it also has a positive impact on their identity, particularly their self-understanding and the way they interact with others [10].

A guide dog can also provide a sense of freedom and independence. In an early German study, 55% of participants reported that a guide dog gave them a greater sense of freedom and independence compared to being accompanied by a human, while another 35% said it depended on the situation [20]. A small interview study (eight participants from across the USA) found that guide dog users reported increased confidence and a feeling that they could achieve personal goals, as well as an increased sense of independence [21]. This often led to more social activity in public spaces. The nature of their social interactions also changed, as they were now seen as guide dog users rather than just blind individuals.

Regular physical activity has well-documented benefits, but many people with disabilities experience significant barriers preventing them from leading an active lifestyle. Specifically, individuals with visual impairments often have a less active lifestyle than the general population but people with visual impairment are more active if they have a guide dog than if not, according to a small Portuguese study [22]. According to an American survey, guide dogs and their humans engage in considerably more walking activity than companion dogs, presumably contributing to health benefits for both human and dog. Off-leash and conspecific activity were found to be similar to that of companion dogs, easing concerns of guide dog welfare in that respect, while the number of human social contacts was higher [23].

An Australian study found that having a guide dog brought both mobility and lifestyle benefits. A guide dog provides a reciprocal relationship that brings structure to daily life, better well-being, encourages social interactions, and makes guide dog users more resilient and independent. Together, these factors give users more freedom and independence in terms of mobility, as well as better mental health. These benefits are inseparable from the dog’s function as a mobility aid, and could be said to constitute an integral part of it [24]. They are, however, dependent on the general attitude to dogs in the country. A South African study found that a guide dog could also be a social repellant, and some friends did not want the dog in their cars [25], which may have reduced transport options. On the other hand, the South African interviewees reported most of the recurring advantages in other studies.

A phone-based survey of 800 Britons, half of which were guide dog users, found that the five greatest benefits of having a guide dog were: easier mobility (81%), more independence (58%), companionship (28%), more confidence (27%), and people being more friendly to them (21%) [1]; largely aligning with the German study [20]. Compared to a white cane, a guide dog is not only a better mobility aid, but also helps to empower, command respect, and enhance the social status of individuals with visual impairments [1]. These psychological and social benefits are quite distinct from those provided by other aids.

However, guide dogs are not unproblematic. Half of the guide dog users in the study by Whitmarsh [1] reported limitations and disadvantages. These included the work involved in taking care of the dog (33%), places where it was inconvenient or not possible to bring the dog (33%), the amount of dog hair (8%), challenges when going on vacation (8%), and unwanted attention from other people (7%). Thus, getting a guide dog involves considerable responsibility and requires adjustments in the user’s daily life and lifestyle to meet the dog’s needs. Moreover, a guide dog is not the right mobility aid for everyone [16], and if users do not have severe enough visual impairment, there is a risk that they may interfere with the dog’s work [1].

Another challenge can arise when the relationship between the user and the guide dog comes to an end. Most users experience grief in this situation. This is because a strong bond also leads to strong emotions, and losing a guide dog may trigger the same feelings as losing a close friend or family member [26,27].

The guide dog’s level of training significantly influences the benefits and disadvantages that users experience [9]. Ultimately, we are dealing with a living being with specifies-specific needs and behaviors—the dog is always a dog, not just a working animal—and substantial differences between individual dogs. The attributes that users most often desire in a guide dog are confidence and safe behavior in traffic. Behaviors that lead to the lowest satisfaction among users are when the dog pulls on the harness or leash, followed by social behavior. The dog’s off-duty behavior can also positively or negatively influence the user’s relationship with their dog, and users differ in attitude to some behaviors. Mobility work is just one aspect of the complex relationship between a guide dog and its user. Therefore, when matching a user with a dog, it is important to take other factors into account [9].

A good match between user and dog is essential because reaping the benefits requires a tremendous amount of trust from the user, who must rely on the dog to perform its tasks correctly [28]. Unfortunately, the relationship is not always successful. In a New Zealandic study of 118 guide dogs, 36% of these were returned before reaching retirement age, which had personal and social consequences for the users [29] and presumably for the dogs. Common reasons for returns include health issues in either the dog or handler, lifestyle changes, and difficulties with the dog’s behavior or training. Factors that characterize a successful relationship between a user and a dog include having the same activity level, the user feeling safe with the dog, the dog being open to new experiences, and similarities in personality, such as how they express calmness, joy, and friendliness [28].

## 2. Materials and Methods

Study participants were individuals in Denmark who had received their first guide dog within the last 5 years. The five-year timeframe was based on the rationale that participants would still have the period without a guide dog relatively fresh in memory, and it is therefore assumed that they are better able to compare that situation to their current one. A recruitment advertisement was created, which the Danish Society for the Blind distributed to their members by email. Six respondents met criteria and were interviewed after giving informed consent.

Data was collected by individual semi-structured interviews, which were conducted by the third author (RH) and took 35–60 min. The interview guide was informed by existing research and included a series of general questions with open formulations to capture the participants’ immediate experiences, opinions, and perspectives. Efforts were made to verify statements, perceptions, and ambiguities continuously during the interviews. Participants were also asked to reflect on guide dog life as seen from their dog’s perspective.

Four interviews were conducted by phone, one via Microsoft Teams, and one physically in the participant’s own home. This flexibility in formats was chosen to ensure access to a larger geographic and demographic audience. All interviews were recorded and transcribed verbatim. Participants are pseudo-anonymized by changing their names, ages, dog names, geographical locations, etc., but given the small community of guide dog users in Denmark, individuals might still be identifiable in the actual transcripts, and these are kept confidential.

An iterative, reflexive thematic analysis approach provided a rich description of the data set and highlighted distinct themes. The study followed the six-step process outlined by Braun and Clarke [30]. The first and third author team familiarized themselves with the data through repeated reading of the transcripts (phase 1). After familiarization, initial codes were generated semantically (phase 2) and sorted into candidate themes and sub-themes (phase 3). Authors reviewed the candidate themes and codes with reference to the data (phase 4). The themes were then refined and named (phase 5) and relevant extract examples and a report was produced (phase 6). The reflexive thematic analysis process was recursive with codes and themes established through a process of moving back and forth across the six phases.

Published criteria for good qualitative research [30] helped to support the analytic rigor of the study. Methods to increase analytic rigor included the checks throughout the interview by summarizing ideas and reiterating participant responses to ensure that the meaning had been correctly understood. Moreover, to strengthen the credibility of data coding and interpretation, two researchers reviewed all transcripts and presented their interpretation and descriptions of themes to the rest of the research team independent of the data collection and analysis [31].

## 3. Results

### 3.1. Presentation of Participants

The six participants of the study varied with respect to several demographic variables, which is a strength for explorative interviews. Age ranged from 33 to 58 years old. Three participants were employed in the labor market, one was a job seeker, and two were on early retirement. Three of the participants lived in an apartment, and three lived in a house. Three participants lived in a bigger city, and three in a smaller city or town. Two of the participants lived alone, two lived with their children, and two lived with their partner and children. While it was the first guide dog for all participants, three of them had had a dog before in their adult life, one had a dog during childhood but not since, and two had never had a dog. All guide dogs were Labrador Retrievers, with five bitches and one male. The length of partnering with the guide dog ranged from one year and three months to four years.

When asked about the reasons for applying for a guide dog, several participants stated that they often walked into things and wished to make it safer to move around:

“So, I was actually getting tired of hurting myself so often. And then I had heard about guide dogs, and how they were a fantastic help, and how it allowed you to get from point A to B quickly, but also that you didn’t use as many resources just getting around” (Interview 6).

Two participants also mentioned that because they are dog lovers, a guide dog was an obvious aid. However, not all participants felt this way. When asked if they were dog lovers, four answered yes, but two participants were not particularly fond of dogs and responded, for example: “*I’ve always been a cat person, and I always thought, I’ll never have a guide dog.*” (Interview 4), but they could understand the need and decided to attend the applicant course to explore the possibilities, which led to the following:

“… me, who doesn’t like dogs, was actually the first one who was lying on the floor with all those guide dogs. Oh, woof, woof, oh, cuddle, cuddle, you’re so sweet! […] So, yeah. Now I’m happy, really happy with Becca” (Interview 4).

Even if the participant initially was not fond of dogs, it seemed possible to develop a bond with a guide dog. When asked whether the realities matched their expectations, all participants reported that it certainly met their expectations—expressed like: *“This is over the hill. I mean, she has exceeded all expectations, and you can multiply that by a thousand.”* (Interview 3) and: *“It’s the best thing I’ve ever done, I think.”* (Interview 2). As the statements above show, all participants were incredibly happy with their guide dogs.

### 3.2. Benefits of Having a Guide Dog

Having a guide dog offers many advantages, both in terms of practical assistance and psychological benefits. Based on the analysis of the interviews, the benefits can be categorized into six subthemes.

#### 3.2.1. Improved Mobility

Improved mobility concerned three perceived benefits: safer movement, less energy spent on moving, and easier movement. All participants mention that they feel it is safer to move around as an advantage of having a guide dog. For example, one participant states:

“I feel much safer. I have a dog that also, in that way, guides me through and looks out for me. I no longer have to worry about walking into things, injuring my eyebrows, or needing stitches at the hospital again, right?” (Interview 6).

Another participant compares this to the time before they had a guide dog:

“I used to hang out on the tracks at [city’s] train station. I once tripped over something because I tried to avoid some people. […] But with the dog, with Karla, she knows, after we’ve made the trip 30,000 times, that she just has to get me off the train. Then she has to find the stairs because there’s a treat waiting for her. […] with the dog, it could really be a matter of life and death.” (Interview 1).

As shown by these examples, the guide dog makes a significant difference in the safety of their human’s movement. This is closely linked to mental well-being, as shown later.

All participants also express that they use less energy to move around since getting their guide dog, and that movement has become easier overall. This contributes to them getting around more, faster, and with less effort, collectively improving their mobility. One additional benefit is that one participant has experienced increased job satisfaction because it is now much easier to get to work.

#### 3.2.2. Impact on Physical Activity Level

Five out of six participants expressed that having a guide dog has contributed to them achieving a higher physical activity level, especially in the form of the walks required when you have a dog. One participant noted: *“Before, I used to skip a lot of exercise, a lot of walks*.” (Interview 3). However, after getting a guide dog, the change was significant: *“I walk with her at least three hours every day. […] I get a lot of exercise now, you could say.”* (Interview 3). This change is linked to the natural need to walk the dog, to the fact that it is easier to get around, and to the next theme of greater freedom and independence.

#### 3.2.3. More Freedom and Independence

All participants express in various ways that their guide dog has given them more freedom. One participant, however, says no to the idea that it has affected their ability to manage more on their own, but states that it requires much less energy. For the others, the impact has been significant in terms of being able to manage more independently. For example, one says:

“She also gives me that freedom to wander off on my own. […] It gives me a little more independence or self-confidence, doesn’t it? […] It’s a really big thing.” (Interview 2), while another states: “A GPS, and Oskar, means I can go to Paris, if I want, alone. […] that must be the biggest advantage, that independence from other people, you could say.” (Interview 5).

The connection between greater freedom and independence is also expressed by another participant: *“You’re much more free to get around, and that creates more independence.”* (Interview 6). As the examples above indicate, this theme is crucial for the participants. Not only by giving them the freedom to get around independently, but also by providing the independence that reduces their reliance on others. This freedom does not just make a difference for the guide dog users, it also has a positive impact on their relatives, especially for the four participants who live with family. Beyond the participants becoming freer and more independent from their loved ones’ help, it also seems to relieve their family members: *“The last few years before I got Becca, I think I used my son a lot, without really thinking about it.”* (Interview 4).

#### 3.2.4. New Social Interactions

All participants mentioned new social interactions. However, whether this is seen as positive or negative change varies, and for several, it is viewed as both an advantage and a disadvantage. The positive aspects of new social interactions are discussed first.

The advantages include the possibility to see old friends more easily: “Now I can go visit my friends and such. I had stopped doing that for a while. I was actually home a lot for a couple of years.” (Interview 2), as well as engaging in many new social interactions because of the dog: “It’s an incredible icebreaker to have a dog like that.” (Interview 1). Another participant shares that the dog is often the opening comment in social situations: “I could hear people just walk by me before. Of course, they did. But now, people greet me. […] It really opened a lot of social doors for me.” (Interview 3). This suggests that the dog acts as a kind of catalyst for new social interactions, bringing them closer to neighbors and fellow dog walkers.

In addition to creating more social interactions, the nature of interactions is also different from before: *“But there’s something about when you walk with your dog. People are friendlier and more chatty.”* (Interview 1). Similarly, another participant expresses experiencing much better understanding and respect from others:

“Now, I’m respected for who I am, and I can quietly ask for help […]. It’s gold to have a guide dog. It has made my life easier. And especially this thing about being met for who I am.” (Interview 4).

Furthermore, two participants mention that more people spontaneously offer them help, even though they needed it more before getting the dog, resulting in more helpful and positive social contacts with others.

While most participants appreciated these new social connections, some noted that the interactions were not always welcome, particularly when they interrupted daily activities or distracted a working dog.

#### 3.2.5. Development of a Special Bond

As previously illustrated, the participants are very fond of their guide dogs. This is also evident in the special relationship they develop over time with their guide dogs. One participant describes it this way:

“I think that’s the most wonderful thing about it. I always say that we share the day, the dog and I […]. It’s a combination of a companion and a helper. It’s invaluable, I think.” (Interview 1). Another participant states that they constantly sense each other’s moods: “It’s strange to call it a friendship, but we’re not two, we’re one […]. I’ve never had that with a dog before. I’ve had dogs, but this is something entirely different.” (Interview 5).

This participant also notes that living together, working together, and doing everything together is likely the reason behind this special bond. Other participants also confirm that it is not just a practical partnership, but a friendship:

“I can honestly say that Nelly is my best friend.” (Interview 2). However, this close relationship also has consequences when they are apart: “She’s really become my extended friend. We’re very close. So, it’s strange if she doesn’t come along.” (Interview 6).

The participants acknowledge that this special bond develops over time and is not necessarily present from the start. One participant, before attending the applicants’ course, was convinced they would not be able to bond with a dog:

“I might not bond with other people’s dogs, but wow, these dogs really are something. And my perception changed very quickly. So, it has been such a strange journey […]. I trust her 100%. We have a fantastic partnership. I just love her […]. We handle everything together, I think. […] It’s like ‘you and me against the world’.” (Interview 3).

So, even if initially skeptical and not feeling a strong connection to dogs in general, it is still possible to develop a special bond with the guide dog. This bond benefits the participants in many ways. For example, four participants express that it becomes easier to handle difficult situations with their guide dog by their side. This deep emotional connection reflects the unique nature of human–animal relationships. The reciprocal nature of this relationship suggests that both species can derive emotional benefits from this relationship.

#### 3.2.6. Better Mental Well-Being

This subtheme covers three perspectives: greater security, better quality of life, and increased happiness, all contributing to better mental well-being.

Participants all mention feeling safer after getting their guide dog, such as: *“It’s huge to be able to feel so free and safe at the same time.”* (Interview 3). Participants express that this sense of security is largely due to feeling safer moving around:

“As I said, it has created a sense of security for me when I’m out and about. I feel much calmer. I feel much safer.” (Interview 6); “It has changed my everyday life. […] On a scale from 1 to 10, I was at 2 before, and now I’m at 10, in terms of security.” (Interview 5)”.

This sense of security also positively impacts other aspects of their lives:

“I’m more confident in what I do. And I need to ask fewer people for help. And that in itself is a huge boost to my quality of life, in terms of independence.” (Interview 6).

All participants report that having a guide dog has improved their quality of life. In the above example, security and greater independence lead to a better quality of life. For another participant, tasks like grocery shopping, which previously required a lot of energy and were difficult to do alone, have become much easier, improving their quality of life: *“Now, I just go to [name of grocery store], ask for help, and then go home. That has really given me a whole new quality of life.”* (Interview 6).

### 3.3. Disadvantages and Challenges of Having a Guide Dog

While the participants had no trouble sharing the benefits of having a guide dog, it was more difficult to identify possible challenges. As one participant stated: *“Well, there really aren’t many.”* (Interview 2). Disadvantages and challenges are discussed together because several participants did not necessarily acknowledge these issues as disadvantages but still expressed that they could be *challenging* in certain circumstances.

#### 3.3.1. Disruptive Distractions

This subtheme comprises two varieties of distraction: the largest category involves disruptions caused by interactions with other people, while the second one refers to other distractions that may disturb the dog during its work.

The dog’s social catalyst effect can be disruptive. It may quickly become distracting when people approach and try to interact with the dog, especially if the participant works in an open-plan office: *“Wow, people came up to pet her, and I almost didn’t have any peace at all.”* (Interview 1). Moreover, it is not just when the dog is off duty that others make contact. All participants report that some people approach the dog while on duty, despite guide dogs wearing a harness during work, which should signal to everyone not to interrupt. This can cause the dog to lose focus, become distracted, and guide less effectively. For some participants, this is more of an issue than for others. While some rarely experience it, others encounter such distractions 3–10 times a day, which can be tiring: *“It’s like, you have to constantly remind people, ‘she’s at work, she’s at work, she’s at work’, I could die from this.”* (Interview 5). The dog’s loss of focus is not only frustrating, but as the participant mentions, it can also be dangerous.

Other distractions mentioned by participants include when people bring their own dogs and allow them to interact with the guide dog, despite the harness that communicates this is a guide dog at work. Additionally, food in public spaces can also pose challenges:

“If there’s a french fry under a seat, she’s definitely going to dive down to get it. No matter if someone is sitting there or not. She’ll go after that french fry. And then I think, ‘now I’ve lost my seat for her’.” (Interview 1).

#### 3.3.2. Difficulty Finding Care

When the conversation shifted to vacations, four of the participants reported no difficulties in finding someone to look after their guide dog. However, for the remaining two participants, this was a definite disadvantage: *“Well, the only downside is… finding someone to take care of her when I can’t.”* (Interview 4). This was particularly an issue when traveling with the person who normally looks after the dog, or when going away for several days to a place or an errand, where taking the dog along was not possible.

#### 3.3.3. More Cleaning

All participants mentioned that there is more cleaning involved, and many noted that there is dog hair everywhere. Four of the participants did not see this as a significant problem, but for two, it was a disadvantage: *“The downside is that the house gets dirtier with her around.”* (Interview 6).

#### 3.3.4. Difficult Start

Participants described the early phase as a major adjustment. This includes practically integrating a dog into one’s life—particularly when visiting people who cannot tolerate dogs, or when staying somewhere that does not allow the dog. Moreover, the process of learning how to walk with a guide dog and adjust to one another:

“You get a fully trained dog, but you still have to find your rhythm, work together—it actually takes six months to a year. […] It was hard work those first 3–4 months. […] If you’re willing to put in the effort, you’ll get ten times back.” (Interview 6).

All participants acknowledged that it takes work in the beginning but stated that it is worth it. However, this adjustment period can also lead to feelings of insecurity:

“In the beginning, many times I thought, ‘Oh, I’m so unsure,’ and I lost my orientation, while she just stood there calmly. What should we do? I have no idea. […] I’d say that the first six months, I was really stressed with her. Because I thought it was hard. […] I was under a lot of pressure, and nervous. I can remember that after six months, I finally started to understand the purpose of having a guide dog.” (Interview 3).

Thus, the adjustment period can be difficult at first. During this time, the user also has to build trust in the guide dog: *“At first, it was hard to let go and just walk with her. […] I had to learn that trust.”* (Interview 2). While such trust was not there initially, all participants expressed that they now have full trust in their guide dogs.

Another aspect that made the initial phase difficult was all the learning involved, including understanding dog behavior, such as: *“How do I read my dog? How the heck do I get her to stop pulling when there are other dogs around?”* (Interview 1). However, over time, all participants reported that these challenges no longer posed any difficulties.

### 3.4. The Dog’s Well-Being

Across all six participants, the dog’s well-being came up as an inherent concern. For instance, if it had been a busy period with a lot of traveling or many visits, one participant described the subsequent period as: *“Then she should be allowed to land, but again, I should too, of course. And then we have a quieter week after that.”* (Interview 4).

Moreover, all participants were aware that their dogs need time off work, which means to have the guide dog harness off and to be just dogs:

“And it’s important to give your working dog free rein, to let them stretch out, run around, and just be a dog.” (Interview 1).

“Well, I really care about her having some free time, and she definitely has that. I mean, she’s often just running around here at home, relaxing, lying in her basket, chewing on a bone or whatever else she’s doing, and sleeping. She’s got a free dog’s life, you know. And as I told you earlier, when you called me, we were out for a long walk. There’s a field near where we live where she can really run around and have fun. We met some other dogs, some of the neighbors’ dogs, and she enjoys playing with them. So, they ran around and played a bit.” (Interview 2).

Regarding the importance of animal welfare, it seems that all participants were aware of the dog’s well-being, particularly considering the dog’s need for a “work–life-balance”. Participants seemed to have an understanding of their dogs’ psychological needs, recognizing signs of fatigue, stress, and contentment. This welfare awareness appears to strengthen the human–animal bond while ensuring sustainable working relationships that benefit both partners.

## 4. Discussion

A Scandinavian welfare state may be a particular context for funding and decisions regarding guide dogs, but the experiences of guide dog users resemble those from other countries.

Advantages associated with having a guide dog, as identified in the analysis, largely align with existing research. These include improved mobility [24], greater ease of getting around [1], and increased levels of physical activity [19,22]. Participants also reported a greater sense of freedom and independence, which is consistent with similar findings from several studies [1,19,20,21,24].

These findings highlight the complex interspecies dynamics inherent in guide dog partnerships, where human well-being and canine welfare are interdependent. The success of these relationships depends not only on the dog’s training but also on the handler’s ability to recognize and respond to their animal partner’s physical and emotional needs.

The finding that a guide dog encourages more social interactions is also in line with previous research [1,21,24]. Additionally, participants reported that these interactions are generally positive, which is consistent with findings from the UK [8] and USA [21]. The mechanism may be a general effect of dogs as catalysts for friendly interaction between strangers [32,33], or a change in attitude to people with observable disabilities when they are accompanied by an assistance dog. Implicit associations tests indicate non-conscious stigma towards people in wheelchairs, which disappears when they are seen with a dog [34], and field studies have found that a person in a wheelchair receives more positive attention from strangers when accompanied by a service dog than when not [35]. While it has not been determined whether the same effect applies to blind people, interviewees in an American study describe the white cane as more stigmatizing than a guide dog [10]. Our study indicates a dog as catalyst effect for guide dogs, but further research is needed to establish its generality as well as specify other mechanisms involved.

A strong bond often develops between the user and the guide dog [1,26], and this is particularly evident in the findings of the current project. The positive impact of having a guide dog on mental health corresponds with results from Australia [24], and regarding quality of life, participants reported improvements that align with a British study [8]. However, it is not possible to determine whether these improvements in mental well-being are due to increased feelings of security, greater emotional well-being, or other factors such as increased happiness. This aspect was not addressed in the reviewed studies, nor was the impact of the guide dog on the user’s family explored.

In contrast, it was more difficult in this project to identify disadvantages compared to advantages. This aligns with the study by Whitmarsh [1], where only half of the participants reported that owning a guide dog came with limitations and drawbacks, but there is limited literature specifically addressing the disadvantages of having a guide dog. The current study’s findings that some participants have difficulties finding suitable care for their guide dogs, as well as the need for increased cleaning, replicate findings in Whitmarsh [1]. There is a divergence between the two studies in relation to the inconvenience of not being able to bring the dog to certain places. In Whitmarsh’s study from the UK, 33% of participants saw this as a limitation, while only one participant in the current study expressed this concern. Additionally, Whitmarsh found that only 7% of participants viewed unwanted attention from others as a disadvantage, whereas half of the participants in the current study reported being bothered by such distractions, which often stem from others’ ignorance about not approaching a working guide dog. These differences may be related to cultural attitudes changing over time or being different across countries, or to the specific ways in which guide dogs are presented to the public. This deserves further study as well as practical attention.

Regarding the adaptation to life with a guide dog, we found that this transition can be difficult. This was also found in an Australian interview study [36], but this theme has not been widely discussed in the literature. One possible explanation for the difficulties could be the way guide dog training is structured. In both studies, individuals received their guide dogs after relatively short handler-dog co-training and must finish training and learning mostly on their own. In comparison, the medical alert and socio-emotional support dogs in Gravrok and colleagues’ study came with more co-training and support (but the physical mobility dogs with even less) [36]. Being quickly left to work independently with a guide dog may exacerbate the challenges during the adaptation period. This seems to be an underexplored theme in research.

Overall, the analysis reveals more benefits than disadvantages and challenges, and the advantages are strongly valued by the participants, all of whom express that they gain significant benefits from having a guide dog. However, this large benefit also creates a certain dependency on the guide dog, which brings its own challenges. Several participants described it as uncomfortable to be without their guide dog, which could be problematic if the dog is injured and eventually the dog will die or no longer be able to serve as an assistance animal. Support systems should include preparation for eventual transitions, grief counseling resources, and assistance with obtaining replacement dogs when needed. A very strong attachment to the first guide dog may also increase the likelihood of Second Dog Syndrome, where a higher percentage of guide dog users return their second guide dog compared to the first dog [29,37]. Retirement issues were not raised in the current study, where participants’ dogs were still rather young, but represents another transition with complex impact, which has been overlooked by research until recently [38].

Another aspect of the participants’ answers is that the guide dog differs significantly from other technologies like lawn mowers or vacuum cleaners. These technologies were designed to solve a small set of specified problems by means of a narrow set of functionalities, e.g., mowing the lawn or vacuuming the floor. A guide dog cannot be reduced to a specific set of functionalities, like getting from point A to point B or getting across the road. This was also discussed by several other studies [1,8,10,21,24,26]: although the functional aspects of getting around are important, the attachment and companionship to a living being is also a main feature.

It could be argued that these added benefits are merely a positive side effect and not relevant for those paying for the dog—whether taxpayers as in this study, or other means of social security. On the other hand, it could also be seen as an integral part of the dog’s support of the handler’s confidence and ability to engage in work and social life; attachment theorists [26] emphasize the usefulness of the “secure base and safe haven” that a bond with the dog can bring. Moreover, it could be seen as an investment in better health for an at-risk group; as studies from other parts of the human animal interaction domain find associations between dog ownership and improved physical health as well as mental well-being [39,40].

### 4.1. Implications for Practice

Findings suggest three areas that may need improvement. First, the challenging nature of the initial adjustment period suggests a need for enhanced follow-up support and resources during the first few months of partnership. This finding is particularly important given that all participants reported significant difficulties during this transition phase. Current practice often leaves new users inadequately prepared, leading to avoidable safety risks and partnership failures.

Thus, we recommend several weeks of initial co-training of the dog-user-pair with trainer assistance, follow-up opportunities over the next year, and a phone-in option of consultancy later.

Second, a backup dog care option when the recipient cannot take the guide dog with them is recommended. In the current study, this was mentioned in relation to vacations but presumably the same need will arise with severe illness or long-term hospitalization of the user. This gap in services can lead to users avoiding necessary medical treatment or losing their guide dogs altogether. Better support for transition and co-training as well as a safety net for dog care under special circumstances might be organized by the original provider of the dog, and we recommend investigation into whether such services could be added to the criteria for good practice of guide dog providers and the funding for a guide dog made to reflect this.

From an animal welfare perspective, guide dog programs should emphasize handler education about canine behavioral indicators and stress management. Regular welfare assessments throughout the dog’s working life could ensure that both human needs and animal welfare remain balanced in these vital partnerships.

The third area of concern is the prevalence of interruptions of guide dogs while they are working. That is disturbing and potentially dangerous, and we recommend public campaigns to make this widely known.

### 4.2. Limitations

While the present study provides insights into the lived experiences of Danish guide dog users and as such adds the first Scandinavian findings to the field, several limitations must be acknowledged. First, the small sample size (*n* = 6). Despite demographic variation in our sample, and although qualitative methods aim for depth rather than breadth, the small participant pool may not capture the full diversity of experiences among guide dog users in Denmark. Second, all participants had their guide dogs for less than five years, which may influence their ability to reflect on long-term challenges or changing relationships with the dog over time. Third, the study relied solely on self-reported data obtained through interviews, which may be subject to recall bias or social desirability bias—especially given the positive views expressed. Fourth, distribution of the invitation though the mailing list of Danish Society for the Blind, who is also a guide dog provider, may have biased our sample toward more satisfied or actively engaged users, potentially underrepresenting negative experiences. Finally, cultural and structural factors specific to our context, such as the welfare system and guide dog training models, may limit transferability of the findings to other countries or settings; however, the number of similarities to findings from studies in other countries suggests otherwise.

## 5. Conclusions

The purpose of this study was to extend the literature on guide dog user’s first-hand experiences by exploring the benefits and challenges seen by guide dog users in Denmark, and to suggest areas of potential improvement. Based on the analysis, it can be concluded that the advantages of having a guide dog include improved mobility, making it easier and safer to navigate, while requiring less energy. Additionally, guide dog users experience a higher level of physical activity, a greater sense of freedom and independence, and enhanced social interactions. Furthermore, a unique bond with the dog is formed, which helps users cope with difficult situations. An important benefit is also improved mental wellbeing, including greater security, more emotional resources, and a better quality of life.

However, there are certain disadvantages and challenges associated with having a guide dog. The study found that these primarily involve disruptive distractions, particularly from other people approaching the dog while it is working, and environmental factors that can disturb the dog’s work. Another challenge is the initial adjustment period, as it can be difficult to adapt to life with a guide dog. For some, it can also be troublesome finding appropriate care for the dog when needed, and there is more cleaning required.

Generally, the study’s findings align closely with existing literature on the subject and identify three areas that could be improved: better support for the transition period, securing a backup dog care option, and increasing public awareness of the need to leave guide dogs undisturbed during work. We recommend that policymakers support public awareness campaigns on appropriate behavior towards guide dogs and enforce quality standards for guide dog “production” that include sufficient processes of matching, training and transition support as well as a backup dog care option.

## Data Availability

Data is unavailable due to privacy and ethical restrictions.
